# How could the quality and outcomes framework (QOF) do more to tackle health inequalities?

**DOI:** 10.1080/17571472.2016.1215370

**Published:** 2016-08-08

**Authors:** Tim Thorne

**Affiliations:** ^a^ Health Care Management iBSc, King’s College London, London, UK

**Keywords:** Quality and outcomes framework (QOF), health inequality, primary care, general practice, public health, payment-for performance (P4P)

## Abstract

This paper outlines how the quality and outcomes framework (QOF) has done little to close the health inequality gap, and proposes possible ways in which future iterations of QOF could do more to address this crucial public health problem.

## Why it matters to me

I feel the quality and outcomes framework (QOF) has been under-utilised as a tool to tackle health inequality in the UK. Now, following the abolition of QOF in Scotland,[1] it finds itself under intense scrutiny. Stimulating debate about QOF’s role in our future NHS, and how this might impact health inequality, is important.

## Key messages

• The latest evidence suggests QOF has not had any significant effect on the health outcome inequality gap
• The process of exception reporting arguably disadvantages those in lower socioeconomic groups
• QOF has shifted focus away from patients and reduced their autonomy
• QOF’s future is in question and debate about its role is of crucial importance
• Modifying QOF to give it more focus on inequality could lead to wide-ranging public health benefits

## QOF under threat

QOF is a primary care payment-for-performance scheme which was launched in 2004.[2] Although it has evolved in the 12 years since, its essence remains the same. It incentivises health care by paying practices for their performance against national evidence-based quality standards in two main areas (clinical and public health).[2] For example, practices receive money according to the percentage of patients over 45 who have had their blood pressure measured in the last five years.

The future of QOF is undeniably uncertain. Since its introduction, the number of clinical indicators has reduced significantly,[3] and across the UK there is steadily growing scrutiny over its role. As of April 2016, QOF is no longer used in Scotland.[4] This follows the Scottish government labelling it as outdated and over-bureaucratic.[1] To ensure quality is maintained, GPs will be expected to work in integrated ‘clusters’.[4] These locally agreed groupings will ensure that every GP is actively involved in collecting data and ensuring quality. This will extend to social and secondary care, and be led by practice and cluster leads. In this system, quality will not be tied directly to funding as it is in QOF. The outcome of this new scheme is certain to have knock-on effects on future iterations of QOF in the rest of the UK.

In England, GPs in Somerset are already working under a locally agreed alternative to QOF.[5] Nationally, the General Practitioners Committee has announced that it is working with the government to look at removing QOF as it stands from a new GP contract.[5] These shifts in the medico-political landscape are against the backdrop of two important and rigorous quantitative analyses. Both have shed considerable doubt on the benefit of QOF. The first, published in the BMJ in 2015, revealed that QOF had not directly led to any decrease in premature mortality rates.[6] The second, published in the Lancet in 2016, found that QOF had not been associated with any significant changes in population-level mortality.[7]

The uncertainty about the future role of QOF in England is important. The potential opportunity for change may provide scope for policy-makers to tackle significant public health problems, such as health inequality.

## QOF’s relationship with health inequality

Spanning from the 1980 Black Report,[8] to the 2010 Marmot Review,[9] evidence showed a widening gap in life-expectancy between the poorest and richest in the UK. Although this gap has narrowed recently,[10] academics have suggested that austerity may undo any positive effects on health inequality.[10] QOF was not introduced with the explicit aim of reducing health inequality.[11] However, there has been much debate on how it may have influenced it.

Looking at the early quantitative evidence, two substantial 2008 studies found QOF to be reducing the health inequality gap.[12,13] However, a more recent and robust analysis from the King’s Fund [11] is useful here. It showed that although practices in areas of varying socioeconomic status score similarly in QOF, there is no evidence that QOF has narrowed the gap in health outcomes. It is possible that the result of the 2008 studies can be explained by the inverse equity hypothesis, which was mentioned by both sets of authors in 2008.

This hypothesis suggests that public health policies are normally accessed first by the rich.[14] There is then an initial widening of the inequality gap before it narrows. One set of researchers argued that this explained why the gap in QOF scores between practices in areas of varying deprivation improved from 6.1% of total QOF score after year 1 to 2.9% after year 2.[12] However, there was a large body of evidence arguing that the benefits seen following QOF may have been due to a pre-existing public health policy.[15] For example, perhaps the rich were already closer to achieving QOF thresholds, leaving more room for improvement in lower socioeconomic groups.[16]

The ‘structural’ theory of health inequality can be used as a framework to analyse this issue. This then helps to understand the more recent quantitative evidence that QOF has not reduced health inequality. This theory attributes health inequality to the latent socioeconomic differences that exist between population groups throughout life.[17]

Firstly, QOF standards do not vary according to the socioeconomic status of the surrounding population.[11] This has led to persistence of the inverse care law,[18] which theorises that health care services are often poorest where most needed.[19] For example, in a thorough analysis, McLean et al. [18] found that QOF did not recognise the extra effort involved in reaching uniform care standards in deprived communities. It was theorised that exception reporting, of challenging or dissenting patients,[20] had contributed to this. This argument is strengthened by recent compelling evidence that deprived patients are more likely to be excluded [21] and that the GP funding system disadvantages patients in deprived areas.[22]

There are also arguments that QOF has shifted focus away from patients and made GPs more paternalistic. Some have suggested that consultations are becoming less holistic and biopsychosocial, and more biomedical.[23] In-depth, qualitative research in primary care has shown that this can lead to a reduction in patient autonomy.[24] Arguably, this further disadvantages those in lower socioeconomic groups who tend to be less engaged in the care process.[25] Evidence that the quality of care for non-incentivised conditions has not improved [26] suggests that those with multiple co-morbidities are likely to be most disadvantaged. As people living in more deprived areas tend to have more co-morbidities,[27] once again it is those in lower socioeconomic groups that may lose out.

QOF’s structure also does little to incentivise GPs to engage in primary prevention.[11] This is likely to most impact deprived communities where there is usually a higher prevalence of chronic disease.[11] Analysing how QOF has impacted the doctor-patient relationship in these ways only strengthens the case that QOF has not improved health inequality.

### How could QOF be re-structured to do more to flatten the social gradient in health?

Although QOF must be recognised for improving performance measurement and data collection, it is now important to think about what it may evolve into, or be replaced by. An important part of this process is to consider how a new GP contract might work as a policy lever against health inequality. Many of the measures outlined below align closely with the NHS Outcomes Framework 2015/16.[28] This framework reflects what the Department of Health feel should lie at the heart of the health system.

Changing QOF standards for each practice according to the socioeconomic status of the surrounding population may help tackle these problems. Authors have proposed systems which reward practices for improvement above an established baseline,[11] or offer differential pricing according to local deprivation rates.[29] This would recognise the extra-effort that is required by practices in deprived areas to meet the same quality standards.[18] This could be tied into future modifications to the global sum allocation (Carr-Hill) formula.[30] This formula is currently under review by NHS England and is also thought to under-represent the needs of deprived populations.[30] Future changes might help to address the problem that working as a GP in a deprived area is harder for a multitude of reasons,[31] many of which cannot be assessed through targets or incentivisation.

QOF’s framework has perhaps been under-utilised as a way to directly reduce health inequality. Effects on inequality have been unintentional rather than a specified aim. To remedy this, QOF could encourage practices to carry out location-specific public health interventions. These include proactive case finding and primary prevention.[11,32] Examples of the latter include incentivising interventions relating to exercise or alcohol intake. This is important due to the higher prevalence of multi-morbidity and unhealthy behaviours in deprived areas.[9] Furthermore, as evidence has shown no link between the size of financial reward and the related health gain,[33] redirecting more funding from process measures towards public health measures may be fruitful. Given the amount of data that is now routinely available to practices,[11] co-ordinating this new approach to target those patients most in need should be achievable.

Removing exception reporting might be a possible step towards health equality, as doctors would be encouraged to adhere to evidence-based guidelines with previously excluded patients.[32] However, the workload and costs of such a move would be extensive. It is also possible that forcing performance measurement of challenging cases would impose unnecessary strain on doctors and patients.[32] Roland [34] has argued that exception reporting is vital, as it allows GPs to exert a degree of personal judgement in the care of patients. A possible solution, recommended in the Marmot Review,[9] is extending QOF thresholds to 100% of patients. This may result in a system whereby deprived patients are less likely to be neglected from QOF registers. This could be a positive step-forward in tackling inequality.

Future iterations of QOF could do more to shift the focus of the consultation back towards the patient. This could be done through incentivising a person-centred approach, with outcome measures meaningful to patients.[35] It has also been suggested that QOF could respond to the needs of local populations through introducing self-reported measures such as quality of life.[3]

QOF could also do more to encourage self-care [6] by moving away from its reductionist approach in managing chronic conditions.[29] This would be especially beneficial for those with multi-morbidity.[36] Changes such as these may improve continuity of care, something that was shown to be damaged by QOF.[37] These factors may also restore high-quality, person-centred care to those most in need. An example of this in action is the proposal for QOF to incentivise a single annual review for patients with multiple conditions.[38] Diverting such focus away from current QOF measures should not overly worry GPs. There is evidence showing that levels of performance do not drop significantly after relevant clinical indicators are removed.[39]

McShane and Mitchell [3] have gone one-step further in proposing a scheme that spans all sectors of the health and social care system. This would give the ability to incentivise every step of the care-process. Such a system would have an unquestionable capacity to influence health inequality nationwide. In addition, the availability and abundancy of data makes this option more plausible than it has been in the past.

On 1 April 2016, Greater Manchester Combined Authority took control of its £6 billion budget, with an explicit aim of reducing health inequality.[40] This will be done through aligning the structures and processes of health and social care with other parts of the public sector such as education and housing. As with the ongoing nationwide trend towards GP federations and super-partnerships,[41] this type of joined-up care and multi-sectoral collaboration could allow payment-for-performance to be managed and controlled more efficiently on a wider scale. This is likely to reduce the bureaucratic burden on overwhelmed GPs, and allow time and resources for positive new approaches to improving health.

Some have advocated abolishing QOF as a way to restore professional independence and re-orientate consultations fully towards patients.[42] Monitoring what happens in Scotland is likely to be helpful in such a debate. Although the fine details of the contract are yet to be announced, the Scottish Government has revealed it will not include tick-box medicine.[43] The new system will instead reward ‘values based quality’.[44] This may encourage GPs to move away from the biomedical nature of QOF, instead relating to ‘issues such as access, continuity, relationship forming over many years, and a holistic approach to all issues impacting on physical, mental and social health’.[44]

In the context of health inequality, this focus on the wider determinants of health at a community level aligns with the visions of the Marmot Review.[9] It will be important to assess how these new measures in Scotland impact health care quality and health inequality. This may help policy-makers when making decisions about the future of QOF in England.

Importantly, it should be recognised that QOF needs to be part of a broader strategy for tackling health inequality and improving whole society health. Incentivisation schemes, like QOF, struggle to recognise diffuse collaborative working that often occurs outside of normal practice boundaries.[36] Adapting QOF in the ways discussed above whilst retaining its framework for data collection and measuring outcomes might be significant. In tandem with complex community-level initiatives such as those underway in Manchester, this could bridge the gap that exists between individual interventions and wider population health. One example could be enhancing the social networks of people in deprived areas to reduce isolation and improve quality of life.

Unfortunately, such changes are unlikely to be problem-free. Issues surrounding who would receive incentivisation payments and who is truly responsible for population-level health may arise. For example, whether the future role of the GP lies more in the consulting room or the community.

## Conclusions

QOF could have done more to reduce the health inequality gap. Now, as QOF nears a crucial juncture, policy-makers could consider using it as a tool to reduce this gap. Measures such as those outlined above may do this and also yield population-level health benefits. Using an updated QOF more efficiently across multi-sectoral working groups, like the one in Manchester, could facilitate this. Consequently, QOF might evolve from performance measurement of individual patients towards having a more complex population-level impact.

## Disclosure statement

No potential conflict of interest was reported by the author.
